# Misdiagnosis of Ocular Adnexal Sporotrichosis: A Case Series

**DOI:** 10.7759/cureus.71657

**Published:** 2024-10-16

**Authors:** Jaslinder Kaur Param Jit Singh, Joanne Shalini Chewa Raja

**Affiliations:** 1 Department of Ophthalmology, Sultan Haji Ahmad Shah Hospital, Temerloh, MYS; 2 Department of Ophthalmology, Universiti Teknologi MARA, Shah Alam, MYS

**Keywords:** fungal culture, histopathological examination, misdiagnosis, ocular adnexal sporotrichosis, oral fluconazole

## Abstract

Sporotrichosis is a subacute or chronic infection affecting subcutaneous tissues, caused by the dimorphic fungus *Sporothrix* spp. This case series discusses three instances of ocular adnexal sporotrichosis treated at a tertiary government hospital in Pahang, Malaysia. It highlights diagnostic challenges, management strategies, and the impact of misdiagnosis and underreporting in the Southeast Asian tropical region compared to other parts of the world. Two of the three cases were initially misdiagnosed, leading to delays in definitive treatment. Incisional biopsies were performed for histopathological and fungal culture analysis, with two cases confirming the presence of *Sporothrix* spp. Following diagnosis, all patients responded well to oral fluconazole, although the recovery duration varied. Ocular sporotrichosis can be misdiagnosed due to its resemblance to other ocular conditions. Prompt diagnosis and early treatment are crucial to accelerating recovery and minimizing the risk of long-term ocular complications.

## Introduction

Sporotrichosis is a subacute or chronic infection that predominantly affects the subcutaneous tissues and is caused by the dimorphic fungi *Sporothrix *spp., including *Sporothrix schenckii*, *Sporothrix globosa*, and *Sporothrix brasiliensis* [1]. Ocular sporotrichosis can manifest in two forms: adnexal, involving the eyelid, conjunctiva, and lacrimal sac, and intraocular, presenting as endophthalmitis [1]. Although increasingly recognized, ocular sporotrichosis remains underreported in tropical Southeast Asian countries, where fungal infections are prevalent [2]. Its clinical presentation often mimics other common ocular conditions, potentially leading to misdiagnosis and delays in appropriate antifungal treatment [2]. This case series, involving three patients from a district government hospital in Pahang, aims to enhance physicians’ clinical awareness and contribute to future epidemiological research both regionally and globally.

## Case presentation

Case 1

**Figure 1:**
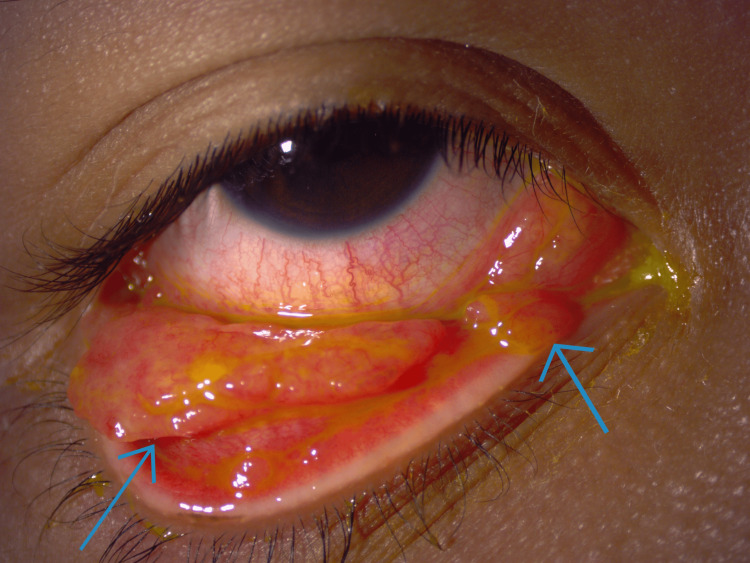
Pedunculated mass (blue arrows) located over the right lower and medial aspect of the palpebral conjunctiva

A 13-year-old female presented to our clinic with right eyelid discomfort and swelling of two weeks’ duration. She denied any history of trauma, feline exposure, insect bites, or sick contacts. Her visual acuity was normal in both eyes, with a 6/6 vision and a normal fundus evaluation. Examination of the anterior segment of the right eye revealed a pedunculated, cauliflower-like mass over the lower and medial aspect of the palpebral conjunctiva, accompanied by a yellowish eye discharge (Figure 1). There was no swelling of lymph nodes or any significant systemic findings.

Initially, she was diagnosed with conjunctival papilloma and was treated with neomycin sulfate, polymyxin B sulfate, and dexamethasone 0.1% ointment, along with oral doxycycline at 100 mg twice daily. However, upon review two weeks later, there was no noted improvement in her symptoms, and the size of the swelling remained unchanged. Due to the lack of response, her treatment was changed to oral fluconazole at 200 mg once daily, along with topical antifungals (amphotericin B and fluconazole eye drops). A biopsy of the mass was performed, and samples were sent for fungal culture and histopathological examination (HPE). Blood work results were normal.

**Figure 2:**
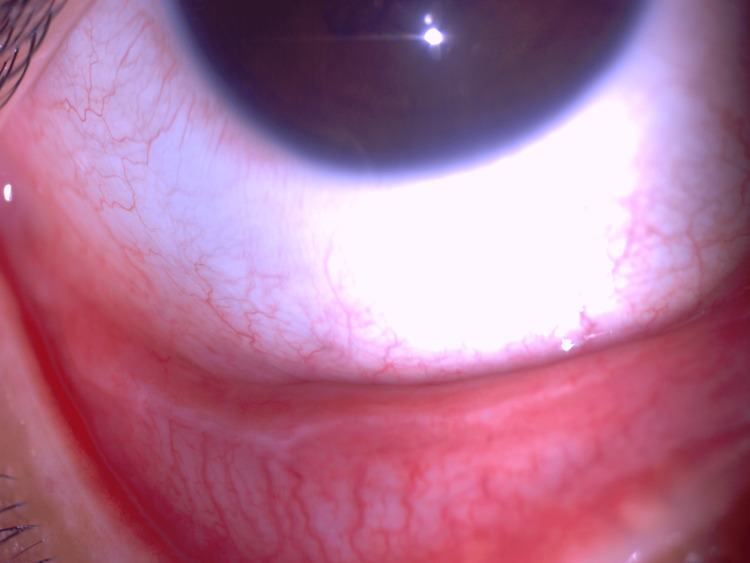
Complete resolution of the conjunctival mass following six months of antifungal treatment

The HPE and cultures from the conjunctival mass were negative for any fungal inoculation. Despite these negative results, her antifungal treatment continued, as there was a clinically good response during subsequent reviews. After six months of antifungal treatment, complete resolution of the lesion was observed (Figure 2), and the patient was discharged from our clinic with no complications.

Case 2

A 28-year-old female presented to our eye clinic with right eyelid swelling and redness of three weeks’ duration. She reported discomfort over her right eyelid but denied any eye discharge, photophobia, or blurring of vision. Upon further questioning, it was revealed that she had a pet cat undergoing treatment for sporotrichosis by her veterinarian.

**Figure 3:**
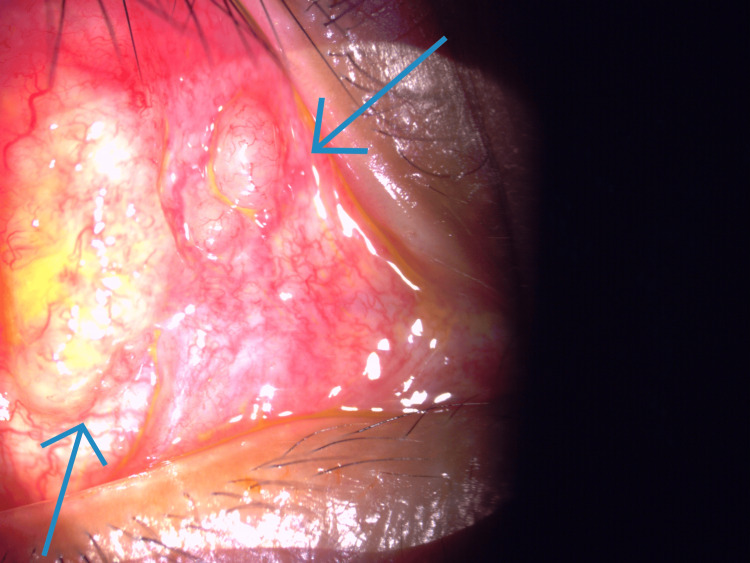
Multiple regular-shaped raised lesions (blue arrows) on the bulbar conjunctiva of the right eye

Examination of both eyes showed visual acuity of 6/6 with a normal fundoscopic evaluation. A slit lamp examination revealed swelling and injection of the nasal bulbar conjunctiva in the right eye, along with multiple raised, regular masses (Figure 3). No lymph node swelling or remarkable systemic signs were observed. Initially, she was diagnosed with pyogenic granuloma and was treated with neomycin sulfate, polymyxin B sulfate, and dexamethasone 0.1% eye drops.

Upon review two weeks later, there was no improvement in the swelling despite treatment. The HPE of the mass revealed granulomatous inflammation, and fungal culture of the tissue was positive for* S. schenckii *fungal inoculation. Consequently, her treatment was changed to oral fluconazole at 200 mg once daily.

**Figure 4:**
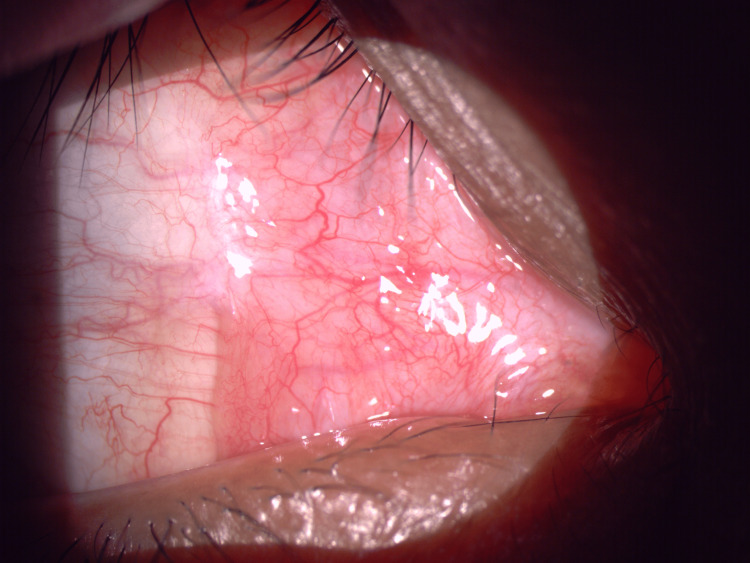
Complete resolution of the lesions following four months of antifungal treatment

Remarkable improvement was noted after four months of treatment, with complete resolution of the lesion (Figure 4), and she was subsequently discharged from our clinic with no complications.

Case 3

A 15-year-old female high school student was referred by her general practitioner for an unresolved right eyelid swelling lasting one month. Prior to this presentation, she had completed a course of oral antibiotics and topical chloramphenicol eye drops for presumed conjunctivitis. She reported no feline contact, trauma, or insect bites.

**Figure 5:**
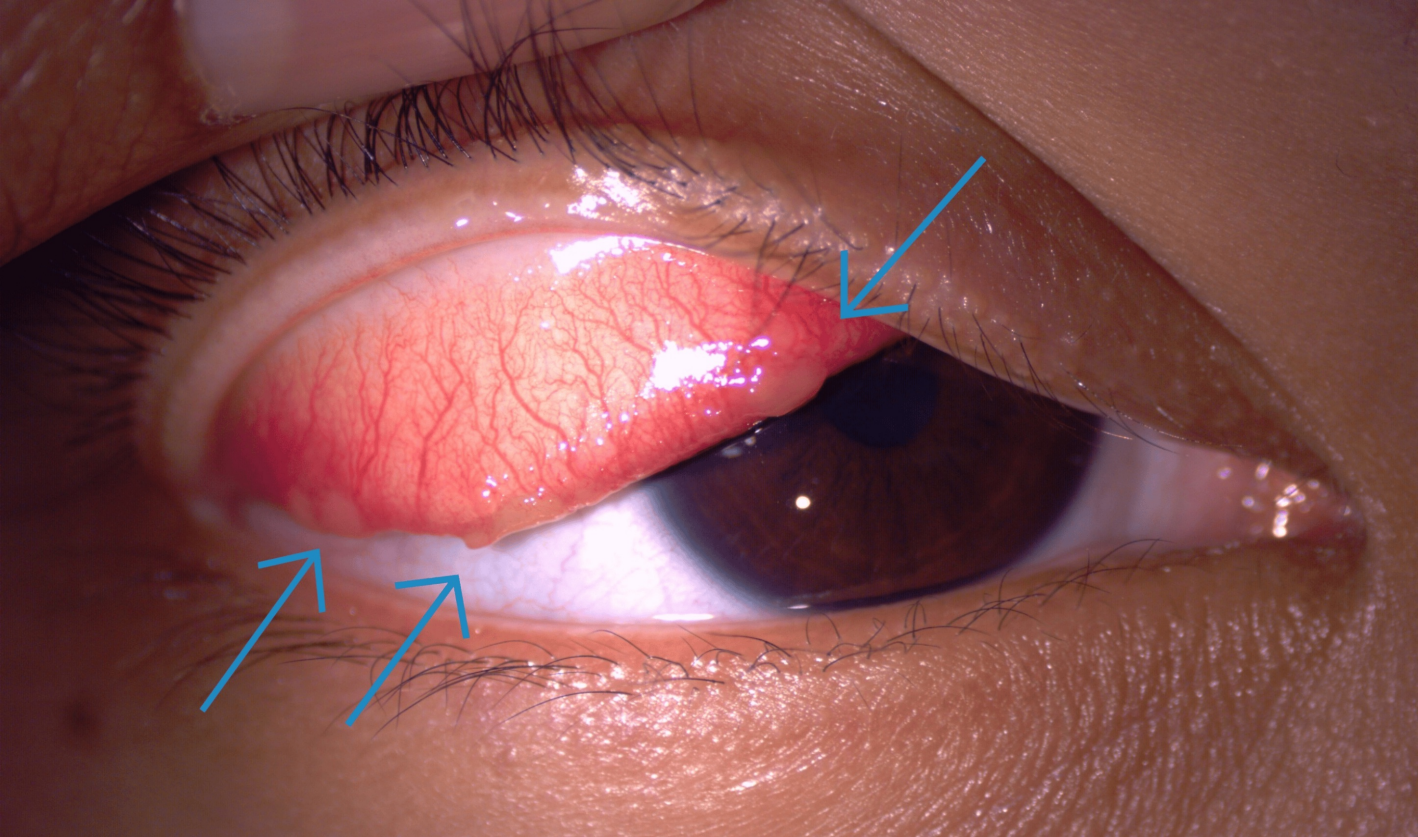
Multiple oval-shaped lesions (blue arrows) on the right upper lid palpebral conjunctiva

Upon review at our clinic, her visual acuity was 6/9 in both eyes, with a normal fundoscopy examination. Examination of the right upper eyelid revealed multiple oval-shaped, raised masses on the palpebral conjunctiva, accompanied by injected conjunctiva (Figure 5). No other systemic signs were noted. Based on the clinical presentation, she was diagnosed with ocular sporotrichosis and initiated on oral fluconazole at a dosage of 200 mg once daily, along with topical antifungals (amphotericin B and fluconazole).

**Figure 6:**
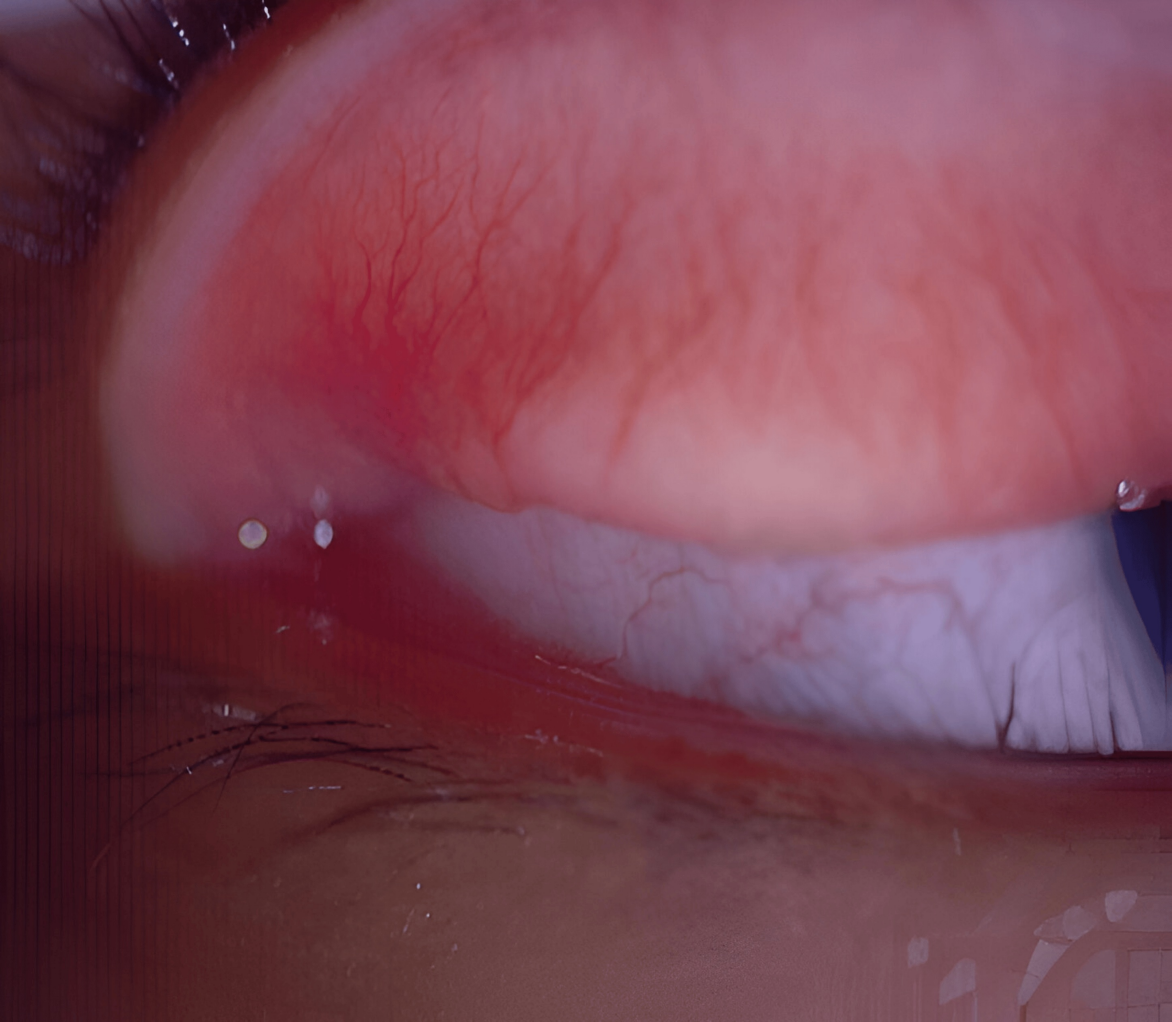
Resolution of the upper lid palpebral conjunctival lesions after completing the three-month antifungal regimen

The HPE of the tissue confirmed *S. schenckii* fungal inoculation; however, fungal cultures showed no growth. Her treatment with oral fluconazole was continued for three months, resulting in a complete resolution of the lesion (Figure 6). She was subsequently discharged from our clinic with no complications.

## Discussion

Sporotrichosis occurs worldwide, with hyperendemicity in regions characterized by warm, temperate, or tropical climates [1,3]. The estimated annual incidence globally is nearly 40,000 new cases, with the majority arising in hyperendemic tropical areas such as Brazil, Peru, Mexico, and China [1,4]. In Asia, cases are sporadically distributed, primarily concentrated in China, with a limited number reported from Japan, Thailand, and Malaysia; this may be due to underreporting [4].

The lymphocutaneous form is the most common presentation of sporotrichosis, while extracutaneous forms are less common but more frequently observed in immunocompromised individuals [2]. Ocular adnexal sporotrichosis can manifest as granulomatous conjunctivitis, Parinaud’s ocular glandular syndrome, or dacryocystitis [3]. Intraocular forms are rare and may present as endophthalmitis, granulomatous uveitis, scleritis, retinitis, choroiditis, or iridocyclitis [1]. Transmission routes for this dimorphic fungus include direct contact or traumatic inoculation from soil, vegetative sources, or organic matter, and rarely, via hematogenous spread [4,5]. Cats are recognized as highly zoonotic transmitters of this fungus, with documented outbreaks occurring in Brazil through scratches, bites, or respiratory secretions [4]. In Jilin, China, factors such as regional humidity, agricultural activities, and histories of trauma have significantly contributed to the 72 reported cases of adnexal sporotrichosis [6]. Several studies have shown a correlation between feline exposure and increased risk of ocular sporotrichosis [2-5,7]. Notably, one of our patients (Case 2) had contact with an ill cat, which likely served as a risk factor for infection.

The variability of clinical manifestations poses a diagnostic challenge, as highlighted in our case series and various reported studies [2]. Two of our cases (Cases 1 and 2) were initially misdiagnosed as conjunctival papilloma and pyogenic granuloma, respectively, due to their clinical similarities, leading to delays in initiating a definitive antifungal regimen. Similar misdiagnoses have been reported in the literature, including a case from Brazil misidentified as bacterial conjunctivitis by Yamagata et al. and a case of granulomatous bulbar conjunctivitis in Malaysia by Mohamad et al., which progressed to legal blindness due to delayed treatment. Additionally, Ahmad-Fauzi et al. reported six cases in Malaysia where misdiagnoses occurred before successful antifungal treatment, while Arinelli et al. noted complications in three out of 26 cases in Brazil due to delays in treatment, including conjunctival symblepharon and fibrosis [2,5,7,8].

The gold standard for diagnosis involves culturing and identifying the causative fungus from clinical samples such as scales, tissue biopsies, or conjunctival mucosal swabs [1]. The “salt and pepper appearance” observed in cultures incubated on Sabouraud dextrose agar is highly indicative of this pathogen [8]. However, the HPE of suspicious lesions is not pathognomonic and may resemble other granulomatous conditions, such as sarcoidosis, cutaneous tuberculosis, foreign body granuloma, and deep fungal infections [9]. Although our patient (Case 2) had a positive *S. schenckii *fungal inoculation from conjunctival cultures, the HPE revealed only granulomatous inflammation, lacking specificity for this dimorphic fungus. Molecular biology methods, such as PCR, offer a specificity of 95% and sensitivity ranging from 3% to 92%, potentially overcoming the limitations of histopathological and direct examinations; however, these methods can be costly and are not readily available in many healthcare settings worldwide [1].

Ocular adnexal sporotrichosis has an excellent clinical prognosis compared to intraocular infections, which may lead to poorer outcomes [1]. Oral itraconazole is the treatment of choice for ocular adnexal sporotrichosis, with a recommended dosage of 100-200 mg per day for adults until complete resolution of the lesions, typically lasting three to six months, followed by an additional two to four weeks after resolution [4,5]. For pediatric patients, the recommended dose is 6-10 mg per kilogram per day, with a maximum of 400 mg per day [3]. Itraconazole exhibits an efficacy profile of nearly 100%, low relapse rates, and fewer side effects, including hepatotoxicity [5,8]. The effectiveness of oral itraconazole for treating ocular adnexal sporotrichosis has been documented in various studies, including 21 cases reported by Ramírez Soto from Peru, three cases by Yamagata et al. from Brazil, and six cases by Ahmad-Fauzi et al. from Malaysia, all treated with itraconazole until complete resolution [2,5,10]. Fluconazole, a synthetic broad-spectrum azole, serves as the second-line treatment option for patients unable to tolerate oral itraconazole [4,8]. In our case series, all patients were successfully treated with oral fluconazole as the first-line treatment due to its availability in our setting. Saturated solutions of potassium iodide, an older therapy available in underdeveloped countries at a lower cost, have been successfully used to treat cutaneous sporotrichosis, including ocular sporotrichosis, although they come with many documented side effects [8,9]. Reinprayoon et al. reported a case from Thailand demonstrating successful treatment of ocular sporotrichosis with topical eye drops of oxytetracycline hydrochloride, polymyxin B sulfate, and dexamethasone rather than the standard oral antifungal regimen [11].

Conversely, amphotericin B is the recommended treatment for systemic, disseminated sporotrichosis, particularly in immunocompromised patients [1]. Liposomal amphotericin B is utilized for systemic sporotrichosis treatment, with caution due to potential renal side effects [1]. Intravitreal and intravenous administration of amphotericin B is employed in cases of endophthalmitis, given the poor intraocular penetration of subconjunctival and oral agents [1].

## Conclusions

Ocular sporotrichosis is a critical differential diagnosis for adnexal lesions. Misdiagnosis can delay the initiation of specific antifungal treatment, thereby increasing the risk of ocular complications. It is essential to exclude ocular mimics and investigate potential risk factors during the diagnostic process. Treatment with oral antifungals has demonstrated significant positive outcomes, leading to the complete resolution of lesions, as illustrated by our case series. Given these findings, a larger-scale epidemiological analysis is recommended to assess the impact of this ocular infection in the tropical Southeast Asian region.

## References

[1] Ramírez-Soto MC, Tirado-Sánchez A, Bonifaz A (2021). Ocular sporotrichosis. J Fungi (Basel).

[2] Ahmad-Fauzi S, Abd-Manan N, Yusof NS, Ibrahim M, Mohamad SA, Muhammed J (2022). Ocular sporotrichosis from a tertiary referral center in Malaysia and review of literature in Southeast Asia. Taiwan J Ophthalmol.

[3] Queiroz-Telles F, Bonifaz A, Cognialli R, Lustosa BP, Vicente VA, Ramírez-Marín HA (2022). Sporotrichosis in children: case series and narrative review. Curr Fungal Infect Rep.

[4] Mohd Rasidin AH, Ang WJ, Raja Omar RN, Ahmad Tajudin LS (2022). Ocular sporotrichosis: different spectrums of clinical manifestations and a review of the literature. Cureus.

[5] Yamagata JP, Rudolph FB, Nobre MC, Nascimento LV, Sampaio FM, Arinelli A, Freitas DF (2017). Ocular sporotrichosis: a frequently misdiagnosed cause of granulomatous conjunctivitis in epidemic areas. Am J Ophthalmol Case Rep.

[6] Zhang Y, Wang Y, Cong L, Yang H, Cong X (2016). Eyelid sporotrichosis: unique clinical findings in 72 patients. Australas J Dermatol.

[7] Arinelli A, Aleixo AL, Freitas DF, do Valle AC, Almeida-Paes R, Gutierrez-Galhardo MC, Curi AL (2020). Ocular sporotrichosis: 26 cases with bulbar involvement in a hyperendemic area of zoonotic transmission. Ocul Immunol Inflamm.

[8] Mohamad SA, Muhammed J, Ibrahim M (2022). Ocular sporotrichosis with limbal stem cell deficiency: an impostor with blinding sequelae. Middle East Afr J Ophthalmol.

[9] Sharma B, Sharma AK, Sharma U (2022). Sporotrichosis: a comprehensive review on recent drug-based therapeutics and management. Curr Dermatol Rep.

[10] Ramírez Soto MC (2016). Sporotrichosis in the ocular adnexa: 21 cases in an endemic area in Peru and review of the literature. Am J Ophthalmol.

[11] Reinprayoon U, Jermjutitham M, Tirakunwichcha S, Banlunara W, Tulvatana W, Chindamporn A (2020). Conjunctival sporotrichosis from cat to human: case report. Am J Ophthalmol Case Rep.

